# Interactive effect between Selenium and Vitamin C levels on risk of hypertension among adult women in the United States: evidence from NHANES 2011 to 2020

**DOI:** 10.3389/fnut.2025.1534535

**Published:** 2025-03-13

**Authors:** Dongfang Wu, Ping Fan, Sze Wan Ching

**Affiliations:** Global Health Research Center, Duke Kunshan University, Kunshan, China

**Keywords:** Selenium, Vitamin C, hypertension, women, NHANES

## Abstract

**Background:**

Hypertension poses an imperative global health risk, affecting over 1 billion people and contributing to cardiovascular disease, especially for women. While previous studies suggest micronutrients such as Vitamin C or Selenium can help reduce blood pressure, research on their interactive effects remains limited.

**Methods:**

This cross-sectional study analyzed data from 9,343 women aged 20 years and older in NHANES (2011–2020). Logistic regression analysis was conducted to evaluate the effect of each micronutrient on hypertension. To account for potential interactions between micronutrients, we calculated the relative excess risk due to interaction, which assessed their combined effect on hypertension.

**Results:**

We confirmed the individual associations of Vitamin C and Selenium with hypertension, showing significant negative correlations (*p* < 0.05). Participants were then divided into four groups, and those with high intakes of both Vitamin C and Selenium had a significantly lower risk of hypertension (*p* < 0.05), supporting the association between the combined intake of these nutrients and lower hypertension risk, though no synergistic effect was observed.

**Conclusion:**

The findings support the combined intake of Vitamin C and Selenium in hypertension prevention, broadening thoughts on the level of nutrition for the treatment of hypertension. These results suggest a potential association between adequate supplementation of Vitamin C and Selenium and lower blood pressure. However, further rigorous clinical studies are essential to validate and strengthen these findings.

## Introduction

Hypertension has been a significant global health challenge for human, affecting approximately 1.39 billion individuals worldwide, it is also the leading modifiable risk factor for cardiovascular disease and early mortality (1). Furthermore, hypertension is the primary factor of death among all cardiovascular disease risk factors. Meanwhile, it is a major contributor to the exacerbation of kidney failure (2, 3). Additionally, hypertension increases the risk of heart disease and stroke, which ranked as the first and fifth leading causes of death in the United States for 2017 (4). Therefore, the adverse consequences of hypertension are notably multifaceted.

Previous research provides substantial evidence on the serious impacts of hypertension. As shown in Table 1, the prevalence of hypertension in the United States varies by age group and gender, with elderly women (aged 65 and above) having a higher prevalence of hypertension, highlighting the importance of early prevention (5). Furthermore, a cohort study found that hypertension and changes in blood pressure during early adulthood were linked to differences in brain volume and white matter in later life, which are associated with neurodegeneration and dementia (6). These results highlight the importance of preventing and managing hypertension in early adulthood.

**Table 1:** Prevalence of hypertension in adults aged ≥20 years from NHANES 2011–2014.

Age group	Male	Female	Difference
20–34	10.7%	7.8%	−2.9%
35–44	23.1%	22.8%	−0.3%
45–54	36.1%	33.2%	−2.9%
55–64	57.6%	55.5%	−2.1%
65–74	63.6%	65.8%	2.2%
≥75	73.4%	81.2%	7.8%

Beyond the significant health implications, hypertension also imposes substantial economic burdens. Compared to normotensive individuals, those with hypertension had approximately 2.5 times the inpatient cost, nearly twice the outpatient cost, and almost triple the prescription medication expenditure (7). Additionally, women may face mounting disease and economic burdens due to hypertension, emphasizing the significance of research in the prevention of this disease.

Studies have shown that certain nutrients can promote the management of hypertension. A re-analysis of National Health and Nutrition Examination Survey (NHANES) data revealed that higher Vitamin E intake was significantly associated with a lower prevalence of hypertension, and another systematic review found that Vitamin D concentration was inversely associated with systolic blood pressure (8, 9). Also, some studies emphasize the importance of Selenium and Vitamin C in preventing hypertension individually. Vitamin C, also known as L-ascorbic acid, is a water-soluble vitamin, which can be obtained either through diet or dietary supplements (10). A meta-analysis has shown that Vitamin C supplementation has significantly reduced blood pressure in subjects with essential hypertension (11). As for Selenium, it is a nutrient, which is essential for the normal functioning of the immune system (12). Furthermore, as a component of glutathione peroxidase, Selenium is a key enzyme in the body’s antioxidant defense system (13). For example, glutathione peroxidase helps maintain nitric oxide in its reduced form and protects against oxidative stress, which suggests that Selenium deficiency could increase the risk of cardiovascular disease (14). On the other hand, dietary Selenium intake has a linear negative correlation with hypertension prevalence in adults (15).

Despite studies exploring the individual effects of these micronutrients, there is a dearth of the interaction of diverse nutrients. This study aims to address the existing research gap by analyzing the interactive effects of Vitamin C and Selenium intake on hypertension and exploring the potential mechanisms among women aged 20 and above. The purpose of our study is twofold. We seek to validate the independent effects of these micronutrients and analyze their interactions, eventually providing new insights to guide nutritional strategies for hypertension prevention.

## Methods

### Study design

This research is a cross-sectional study utilizing data from NHANES, spanning from 2011 to 2020. NHANES, initiated in the early 1960s, is a long-standing research program aimed at evaluating the health and nutritional status of adults and children in the United States, enhancing the wider dissemination of perception of public health. The survey includes interviews that cover a range of topics, including demographic, socioeconomic, dietary factors, and health-related issues such as the history of hypertension and intake of trace elements (16). This extensive database indisputably provided imperative evidence to support our exploration of the interaction between Vitamin C and Selenium concerning hypertension in women.

### Participants

**Figure 1:**
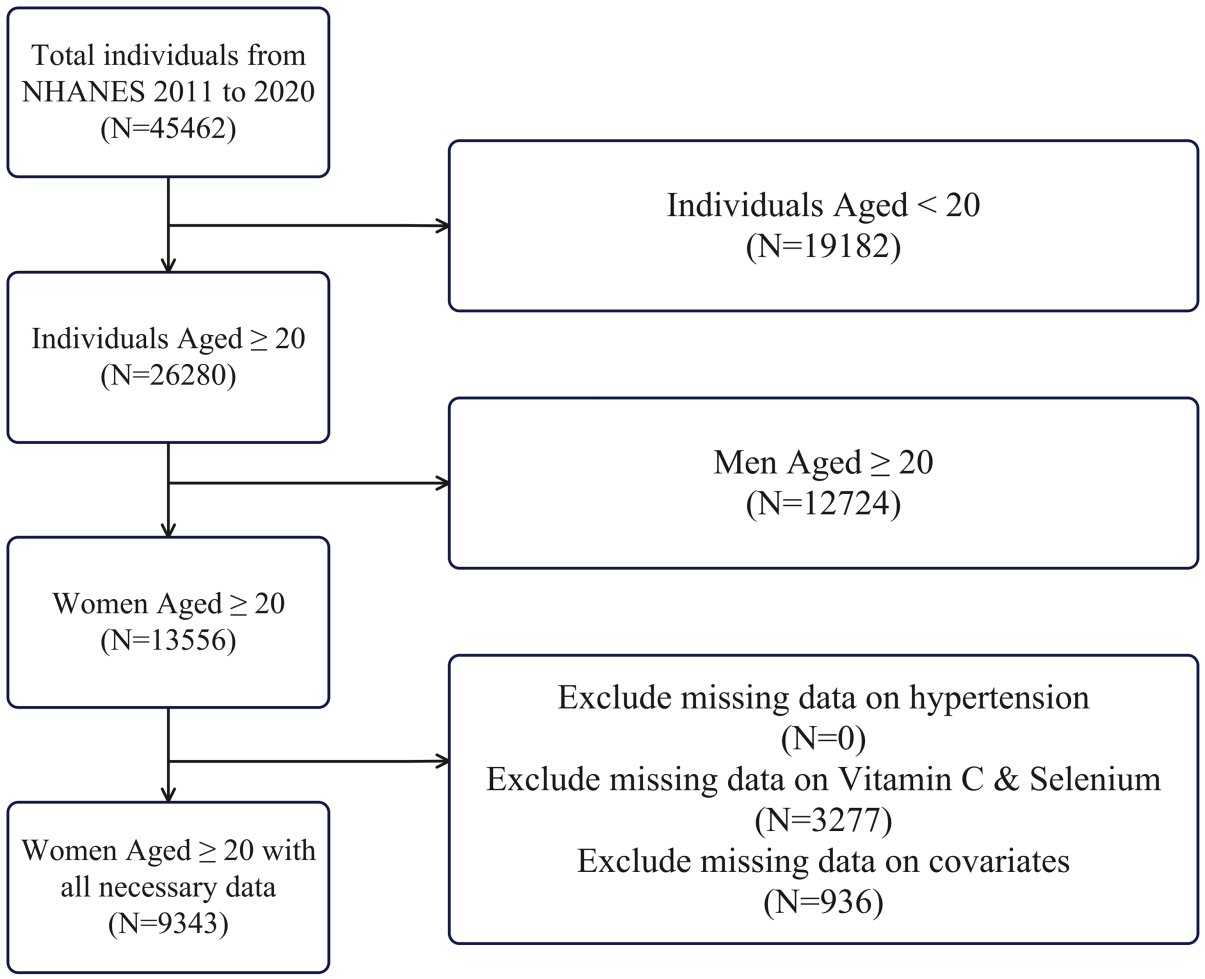
Flow process of participant selection.

As illustrated in Figure 1, we focused on women aged 20 years and older who had complete data for blood pressure measurements, dietary intake (including Vitamin C and Selenium), relevant demographic variables (age, race, education, and income), and behavioral factors (alcohol use, caffeine intake, sodium intake, and recreational activities). Exclusion criteria included missing or implausible dietary data and absence of valid blood pressure measurements, and 9,343 participants were retained for the final analysis.

### Data collection

Blood pressure was measured using standardized procedures, and hypertension was determined if participants met at least one of the following criteria: (1) systolic blood pressure ≥ 140 mmHg or diastolic blood pressure ≥ 90 mmHg, (2) told by a doctor or other health professional that have hypertension, and (3) current use of prescribed medicine for hypertension. Vitamin C, Selenium, caffeine, alcohol, and sodium were assessed by two days and determined by averaging the consumption of participants during this period.

### Statistical analysis

We first conducted descriptive analyses to summarize participants’ demographic and behavioral characteristics using proportions. To assess the individual associations of Vitamin C and Selenium intake with hypertension, we performed logistic regression with three models that incrementally adjusted for demographic variables (Model I), socioeconomic and behavioral variables (Model II), and dietary variables (Model III). Odds ratios (ORs) and 95% confidence intervals (CIs) were estimated to quantify these associations. For the interaction analysis, participants were categorized into four groups based on whether their Vitamin C and Selenium intakes met the recommended dietary intake levels, and we calculated the relative excess risk due to interaction (RERI) to evaluate the combined effect of these micronutrients on hypertension. A *p*-value of less than 0.05 was considered statistically significant.

## Results

### Characteristics of participants

As indicated in Table 2, a total of 9,343 women aged 20 years and older were included in this study and divided into four groups based on whether their Vitamin C and Selenium intakes met the recommended: 75 mg/day of Vitamin C and 55 mg/day of Selenium for adult women, as outlined by the Dietary Reference Intakes from the Institute of Medicine, Food and Nutrition Board. Group 1 included women whose intakes of both Vitamin C and Selenium were below the recommended levels. Group 2 included those with Vitamin C intake above and Selenium intake below the recommended levels. Group 3 consisted of participants with Vitamin C intake below and Selenium intake above the recommended levels. Group 4 encompassed women whose intakes of both Vitamin C and Selenium were above the recommended levels.

**Table 2:** Weighted characteristics of the participants.

Variable	Total *N* = 9,343	Group 1 *N* = 854	Group 2 *N* = 4,732	Group 3 *N* = 364	Group 4 *N* = 3,393	*p*-value
Age group (%)						**0.000**
≤50 years	4,858 (52.00)	401 (46.96)	2,553 (53.95)	154 (42.31)	1,750 (51.58)	
>50 years	4,485 (48.00)	453 (53.04)	2,179 (46.05)	210 (57.69)	1,643 (48.42)	
Race (%)						**0.000**
Non-Hispanic White	3,666 (39.24)	365 (42.74)	2,013 (42.54)	121 (33.24)	1,167 (34.39)	
Others	5,677 (60.76)	489 (57.26)	2,719 (57.46)	243 (66.76)	2,226 (65.61)	
Education level						**0.000**
Less than high school	1,635 (17.50)	217 (25.41)	809 (17.10)	75 (20.60)	534 (15.74)	
High school	1,975 (21.14)	208 (24.36)	1,111 (23.48)	72 (19.78)	584 (17.21)	
More than high school	5,733 (61.36)	429 (50.23)	2,812 (59.43)	217 (59.62)	2,275 (67.05)	
Recreational activities (%)						**0.000**
Yes	3,918 (41.94)	308 (36.07)	1,823 (38.52)	156 (42.86)	1,631 (48.07)	
No	5,425 (58.06)	546 (63.93)	2,909 (61.48)	208 (57.14)	1,762 (51.93)	
Income (%)						**0.000**
<5	7,730 (82.74)	757 (88.64)	3,971 (83.92)	315 (86.54)	2,687 (79.19)	
≥5	1,613 (17.26)	97 (11.36)	761 (16.08)	49 (13.46)	706 (20.81)	
Alcohol (%)						**0.000**
<5	7,534 (80.64)	730 (85.48)	3,801 (80.33)	309 (84.89)	2,694 (79.40)	
≥5	1,809 (19.36)	124 (14.52)	931 (19.67)	55 (15.11)	699 (20.60)	
Caffeine (%)						**0.000**
<120	5,804 (62.12)	556 (65.11)	2,730 (57.69)	281 (77.20)	2,237 (65.93)	
≥120	3,539 (37.88)	298 (34.89)	2,002 (42.31)	83 (22.80)	1,156 (34.07)	
Sodium (%)						**0.000**
<3,000	5,417 (57.98)	835 (97.78)	2,601 (54.97)	353 (96.98)	1,628 (47.98)	
≥3,000	3,926 (42.02)	19 (2.22)	2,131 (45.03)	11 (3.02)	1,765 (52.02)	

Bold values indicate statistical significance.

After conducting a chi-square test, which is used to assess whether the distribution of a given categorical variable (e.g., age group, education level) differs significantly among the four groups (Group 1 to Group 4), we found that the demographic distribution was relatively balanced across different characteristics, as shown in Table 2, with the data rounded to two decimal places. For example, about 52.00% of participants were aged 50 years or younger. Regarding ethnicity, 39.24% of participants were Non-Hispanic White. In terms of education, 38.64% had up to a high school education.

### Association of Vitamin C and Selenium individually with hypertension

**Figure 2:**
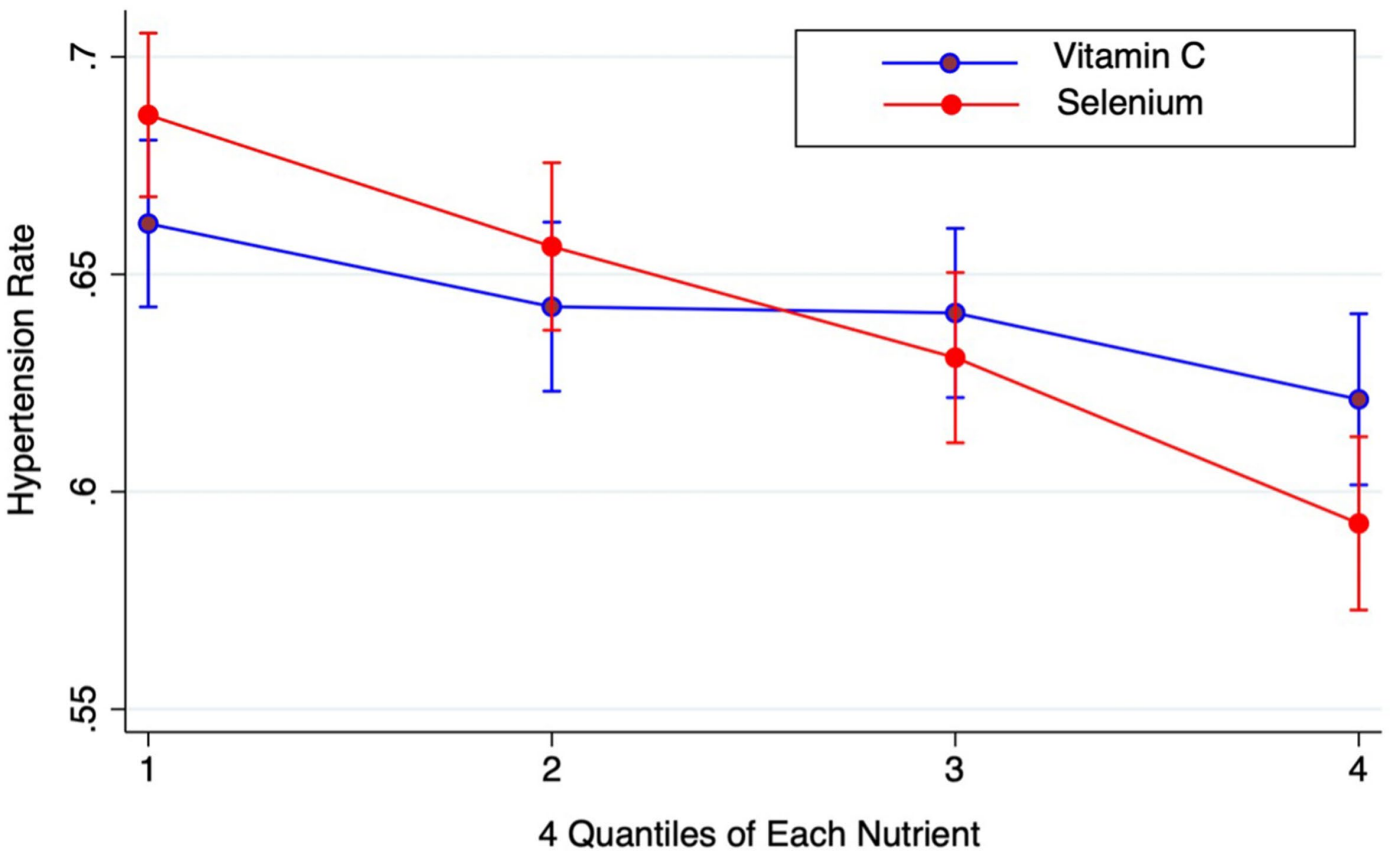
Hypertension rate by quartiles.

The individual effects of Vitamin C and Selenium on hypertension serve as a critical foundation for our investigation of their interaction. First, we validated previous research on the independent relationships between hypertension and Vitamin C, as well as Selenium, using participants’ data. The results were consistent with the prior findings that Vitamin C and Selenium have positive effects on preventing hypertension for the percentage of hypertension by quartiles of each nutrient exhibits a negative correlation, as shown in Figure 2.

Table 3 presents the results of the logistic regression analysis for these independent relationships. It shows that in all three models, both Vitamin C and Selenium had *p*-values below 0.05, indicating statistically significant associations with hypertension. Additionally, the odds ratios suggest a slight negative correlation between these two nutrients and hypertension.

**Table 3:** Association of Vitamin C and Selenium individually with hypertension.

	Model I			Model II			Model III		
	Odds ratio	95% CI	*p*-value	Odds ratio	95% CI	*p*-value	Odds ratio	95% CI	*p*-value
Vitamin C	0.999	0.998,0.999	**0.000**	0.999	0.998, 1.000	**0.001**	0.999	0.998, 0.999	**0.000**
Selenium	0.999	0.997,1.000	**0.006**	0.999	0.998, 1.000	**0.033**	0.998	0.997, 1.000	**0.025**

Model I: adjusted for age, race.

Model II: adjusted for recreational activities, education level, income on the base of Model I.

Model III: adjusted for alcohol, caffeine, sodium on the base of Model II.Bold values indicate statistical significance.

### Association between the interaction of Vitamin C and Selenium with hypertension

To analyze the interactive effect of Vitamin C and Selenium with hypertension, we categorized participants into four groups based on the recommended intake levels of these two nutrients. The interaction between these groupings and hypertension was then analyzed using the RERI method, with the results shown in Table 4.

**Table 4:** Interactive analysis.

	ERR	EIM Std.err.	95% CI	*p*-value
Group 2	0.080	0. 043	−0.000,0.167	0.051
Group 3	−0.043	0.025	−0.091,0.006	0.085
Group 4	−0.100	0.024	−0.147,-0.051	**0.000**

Group 2: Vitamin C intake is above the recommended value, Selenium intake is below the recommended value.

Group 3: Vitamin C intake is below the recommended value, Selenium intake is above the recommended value.

Group 4: Vitamin C intake is above the recommended value, Selenium intake is above the recommended value.

Notably, the *p*-value for Group 4 is well below 0.05, indicating that the result is statistically significant, which provides statistical support for the role of combined Vitamin C and Selenium intake in association with a lower risk of hypertension. However, from the perspective of excess relative risk (ERR), the value for Group 4 is close to −0.1, indicating that when both Vitamin C and Selenium intake exceed the recommended levels, the effect of their combined intake could be less beneficial or even worse than the individual intake of each nutrient concurrently.

## Discussion

While previous studies have examined the independent effects of Vitamin C and Selenium on cardiovascular health, there has been limited research on investigating their combined interaction effect on hypertension. This study aims to bridge this research gap by analyzing how the interaction of Vitamin C and Selenium intake affects hypertension among women in the United States using NHANES data from 2011 to 2020.

This research demonstrates that women with Vitamin C and Selenium intake individually showed significantly lower risk of developing hypertension compared to those with deficient intake of the nutrients. The result notably supports previous studies on the correlation between the intake of Vitamin C and Selenium on hypertension separately. Furthermore, it articulates that the interaction of Vitamin C and Selenium is associated with lower blood pressure among women in the United States, although no synergistic effect was observed and its causation cannot be definitively established.

Our findings both support and extend previous research on the role of Selenium in human health, which received less attention compared to Vitamin C. In the early 1990s, research showed that there is an association between serum Selenium levels and cardiovascular disease in populations that exhibit low Selenium concentrations (17). A study in 2007 found that consuming more foods rich in Selenium could have positive effects on human health, especially cancer prevention (18). In 2012, a study on Selenium and human health found that when Selenium is integrated into selenoproteins, it exerts a variety of pleiotropic effects, including antioxidant and anti-inflammatory actions, as well as the production of active thyroid hormones (19).

Nevertheless, most of the studies reviewed the relationship between Selenium consumption and human health. The interaction of other nutrients and their mechanisms still needs to be further explored. For example, a previous study concluded that the elevated blood pressure and higher prevalence of hypertension at high Selenium levels observed in the NHANES 2003–2004 study align with earlier research (20). It is important to note that high Selenium levels can have toxic effects, potentially leading to adverse health outcomes including selenosis, gastrointestinal disorders, and neurological complications which highlights the complexity of its role in human health (21). The interaction between diverse nutrients and Selenium on specific diseases still lacks enough attention from scholars, which remains an area that requires further accelerated expedition in the future.

Several limitations should be considered when interpreting these results. From the perspective of data transparency, this study obtained data from NHANES between 2011 and 2020, which consists of unilateral state samples, with certain demographic groups or geographic regions are underrepresented or excluded from the survey, potentially causing a dearth of validation and pervasiveness worldwide. In addition, the accuracy and reliability of some self-reported data like health behaviors, dietary intake, and blood pressure in NHANES may be affected by recall bias or social desirability bias, which could lead to misclassification or underestimation of the true associations between nutrients and hypertension. Moreover, the use of cross-sectional data from NHANES restricts the ability to establish causal relationships between the intake of Vitamin C and Selenium and hypertension among the life course population in the United States, making infants, children, and adolescents neglected. Furthermore, while the large sample size provides statistical power, it may also detect very small effects that, although statistically significant, might not be clinically meaningful or could be due to chance rather than true associations.

## Conclusion

In conclusion, our findings provide evidence that the combination of adequate Vitamin C and Selenium intake is associated with lower hypertension risk among U.S. women, suggesting that considering nutrient interactions, may be crucial for understanding and managing hypertension. This highlights the need for a more comprehensive approach to studying nutrient interactions in disease prevention, including their potential synergistic or antagonistic effects.

Future research should focus on longitudinal studies to validate these results and explore the underlying mechanisms of interacted effect between these nutrients and hypertension. Such studies would also benefit from including diverse populations to enhance the generalizability of the findings and provide clearer guidelines for hypertension prevention through nutrition.

## Data Availability

The original data presented in the study can be found at https://wwwn.cdc.gov/nchs/nhanes.
